# Supplementary material for: Vibrio cholerae O139 genomes provide a clue to why it may have failed to usher in the eighth cholera pandemic
Source: Nat Commun. 2022 Jul 5;13:3864. doi: 10.1038/s41467-022-31391-4 (PMC9256687; doi:10.1038/s41467-022-31391-4)
Supplement: Supplementary file 1 — Supplementary Information [file 41467_2022_31391_MOESM1_ESM.pdf]

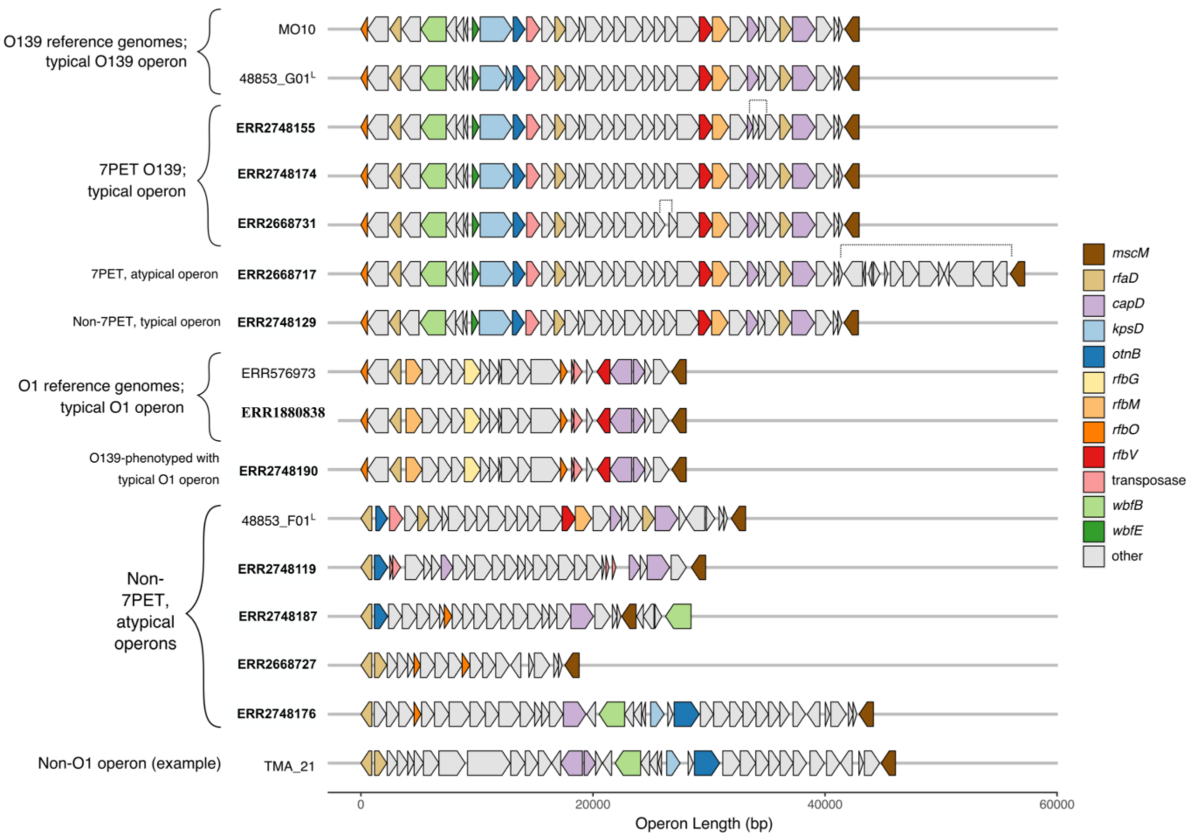

S. Figure 1: Variation in the gene order and arrangement of the O139 operon within our dataset, compared to selected O139, O1 and non-O1/O139 references. Gene homology was based on HMM models and gene arrangement was gleaned from the annotated assemblies. Genes are coloured by function (see key). Some variations in the arrangement of the typical O139 operon are highlighted with faint brackets. Genomes from our dataset are denoted by bold font. All genomes were sequenced with short read technology, except for those denoted by L, which were sequenced using long read technology. For simplicity, only one or two examples of each variant are shown.

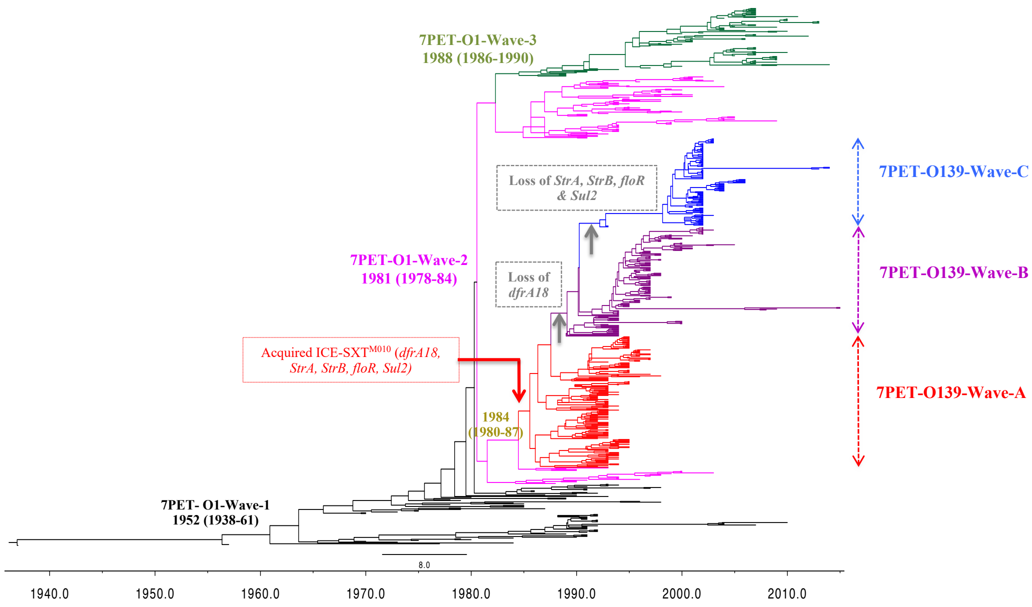

S. Figure 2: Bayesian time-measured phylogeny of 7PET including the O139 sublineage. Highlighted dates represent the most recent common ancestor (MRCA) shared by the *V. cholerae* O1 waves (1-3) and O139 waves (A-C) as median values predicted by BEAST with 95% confidence intervals on the estimated date. The dotted lines indicate the entire O139 lineage with three waves marked as wave A, B and C. Three important events of ICE SXT acquisition to successive AMR loss have been marked with arrows.

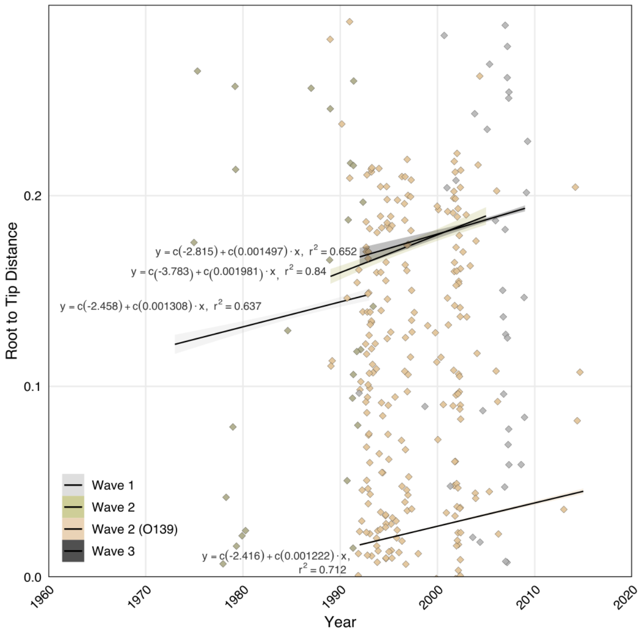

S. Figure 3: Evolutionary dynamics of *V. cholerae* O139 in the context of *V. cholerae* 7PET transmission waves. Linear regression plot of the relationship between the RTT and year of isolation, i.e. the SNP accumulation rate of each of the O1 transmission waves, and the O139 sublineage. Points are coloured by wave (see key). R^2 values and equations of the lines are also shown in the plots. The mean RTT is depicted by the linear regression line, with standard error depicted by the shaded bars. R2 values and equations of the lines are also shown in the plots.

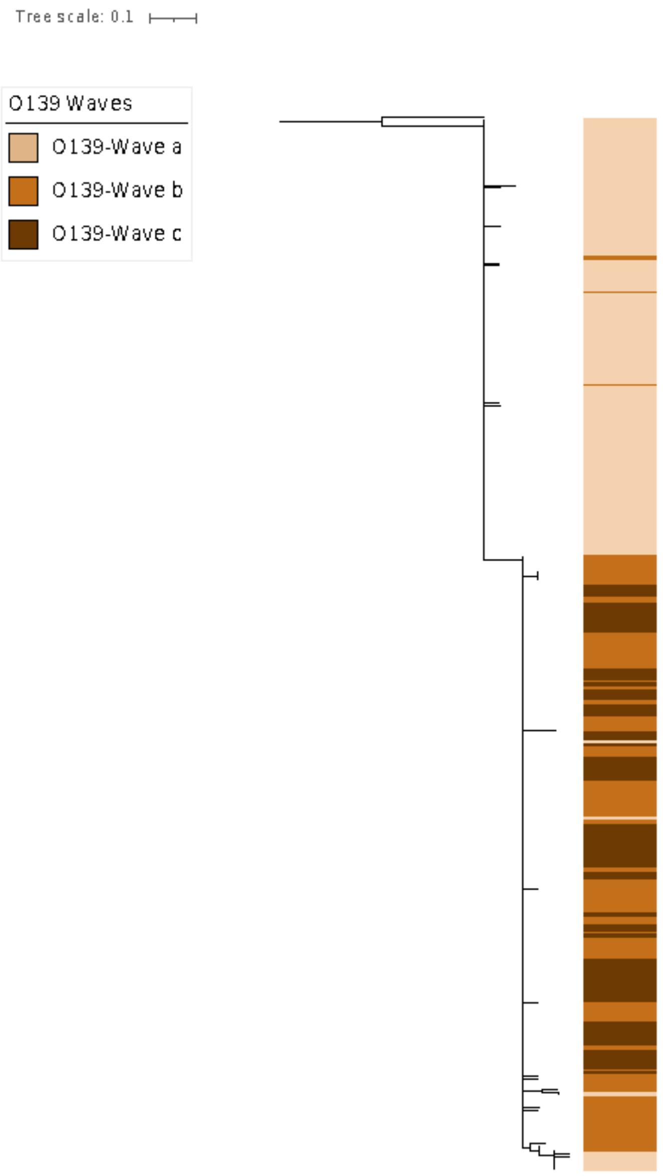

S. Figure 4: Maximum likelihood phylogenetic tree of the core ICE SXT. *V. cholerae* O139 ICE SXT element analysis constructed with 36 SNPs from a whole alignment of 51,961 bases against the reference AY055428. Each of the O139 waves-A to C are represented as a coloured strip (see key).

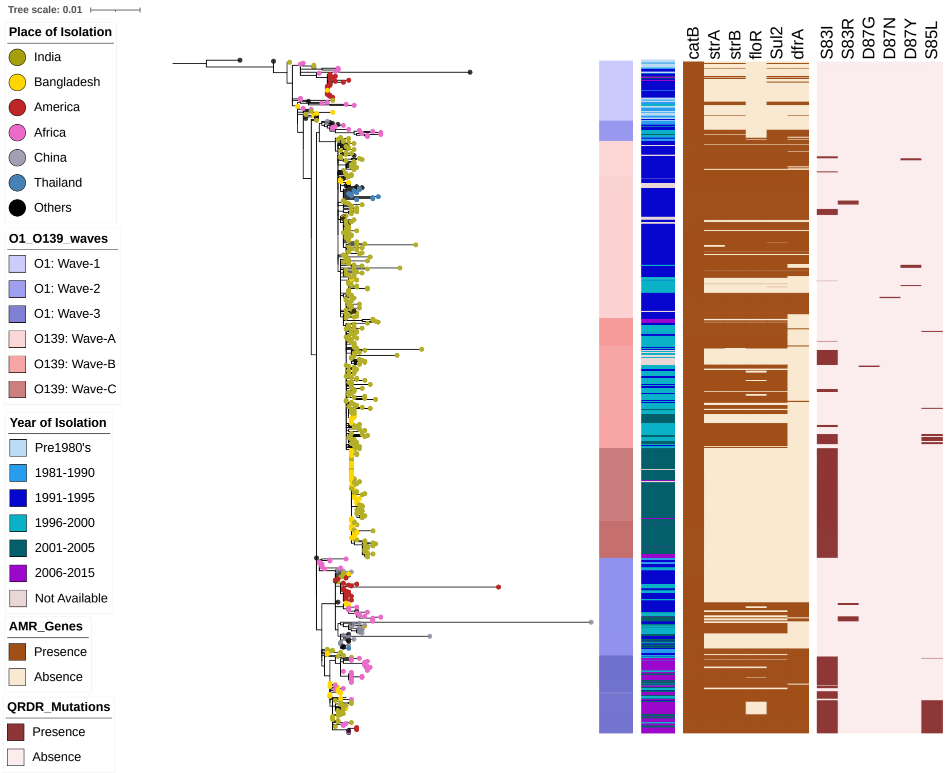

S. Figure 5: Maximum likelihood phylogenetic tree of the 7PET O1 and O139 sub-lineage. Place of isolation was labelled as colored branch tips. Color bar indicates the O1 wave, serogroups and year of isolation. Presence/Absence of antimicrobial resistance genes and the chromosomal QRDR mutations (*GyrA*-S83I, S83R, D87G, D87N, D87Y; *parC* – S85L) are labelled for 7PET O1 and O139 strains. Scale bar represents the number of substitutions per site per genome

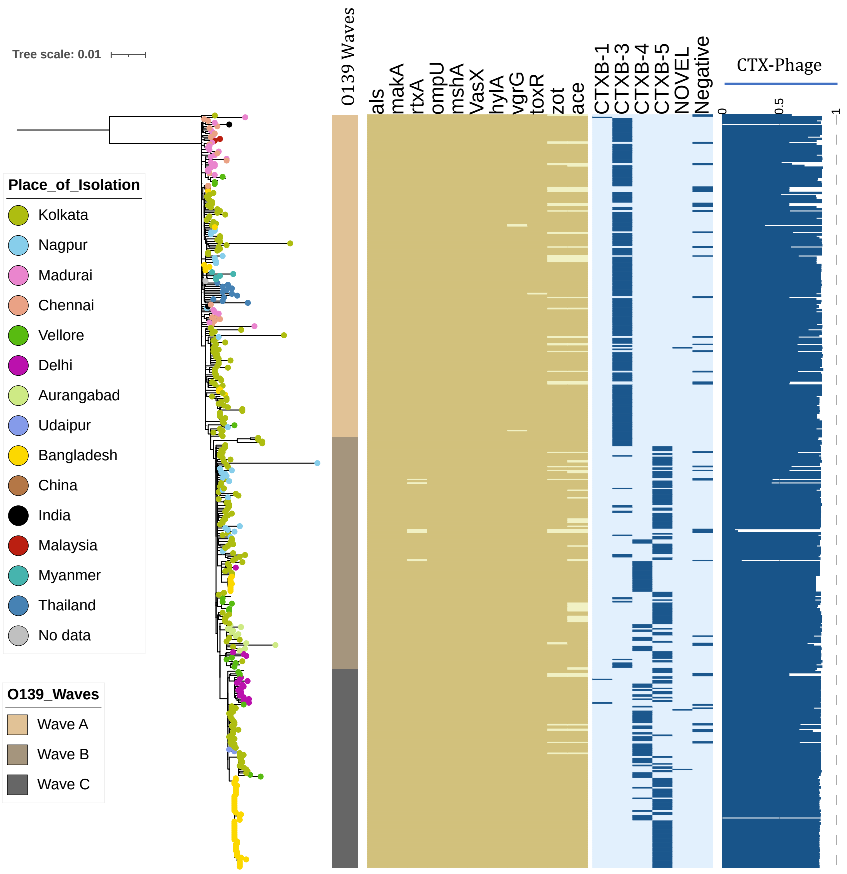

S. Figure 6: Virulence gene distribution among *V. cholerae* 7PET O139. Maximum likelihood phylogenetic tree of the *V. cholerae* 7PET O139 sub-lineage, constructed from whole genome SNPs relative to the *V. cholerae* H01 long-read reference genome. Tree tips are coloured by place of isolation (see key). Colour bars depict the O139 clade/wave (see key), whilst gold and green heatmaps represent presence/absence of various virulence factors and vibrio pathogenicity islands, respectively (see key). CTX phage bar indicates the percentage presence of the entire CTX-phage region (24,248 bp) of O139 strains mapped against N16961 region (VC_1451 to VC_1465) on a scale of 0 to 1. Scale bar represents the number of substitutions per site per genome.

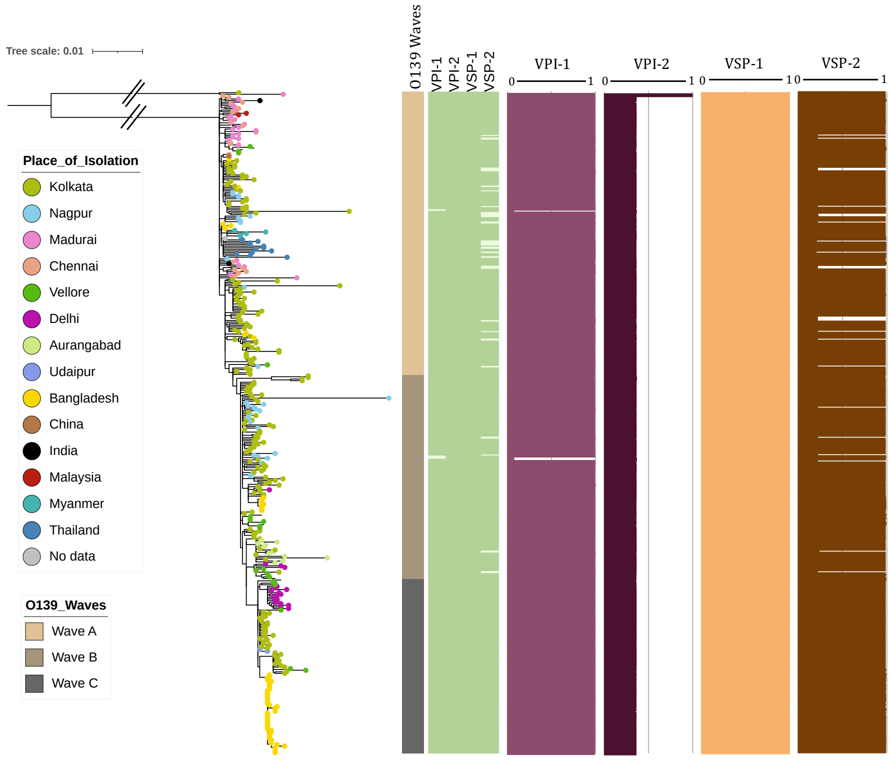

S. Figure 7: Maximum likelihood phylogenetic tree of the 7PET O139 sub-lineage. Color bar indicates the O139 waves (wave A-C). Heatmap represents the presence/absence of VPI-1 (VC_0817 to VC_0847: 40883 bp), VPI-2 (VC_1758 to VC_1809: 56770 bp), VSP-1 (VC_017 to VC_0186: 16679 bp) and VSP-2 (VC_0489 to VC_0517: 31651 bp) islands. Color bar indicates the percentage presence of the entire regions of VPI-1, VPI-2, VSP-1 and VSP-2 islands of O139 strains mapped against N16961 region on a scale of 0 to 1. Scale bar represents the number of substitutions per site per genome.

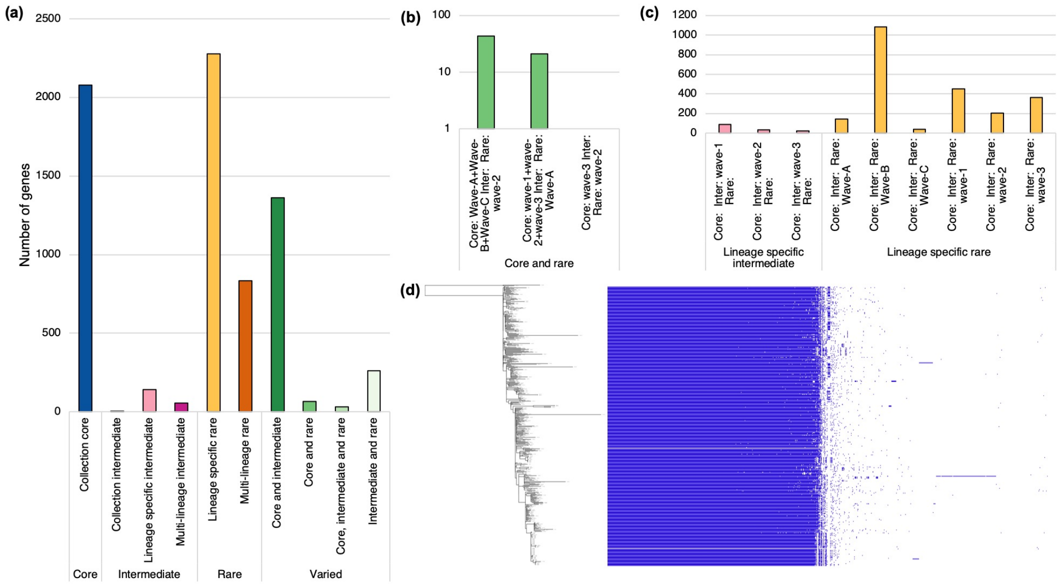

S. Figure 8: Pan genome analysis of *V. cholerae* O139. **(a)** Distribution of genes among different gene categories, as classified using the software Twilight after clustering using Roary. **(b)** Number of genes core to some groups but rare to others ("varied frequency" among groups). **(c)** Number of lineage-specific intermediate frequency or rare genes. **(d)** *V. cholerae* O139 pan genome matrix visualised against the *V. cholerae* 7PET O139 subtree. Blue blocks represent genes.

Supplement Table 1: Genome characteristics of non-7PET O139 serogroup *V. cholerae*

Marker genes		22776_8#124	22776_8#104	23385_1#115	23385_1#47	23385_1#57	23385_1#160	23385_1#52
Species	***ompW***	+	+	+	+	+	+	+
Serogroup	***wbfZ_O139***	-	-	-	-	+	+	+
7PET	***VC2346***	-	-	-	-	-	-	-
Virulence	***ace***	-	-	-	-	-	-	-
	als	+	+	+	+	+	+	+
	chxA	-	+	-	-	-	-	-
	ctxA	-	-	-	-	-	-	-
	hlyA	+	+	+	+	+	+	+

	ompT	+	-	-	-	-	-	-
	ompU	+	-	-	-	-	-	-
	makA	+	-	+	-	+	+	+
	mshA	+	+	-	-	-	-	-
	rtxA	+	+	+	+	+	+	+
	toxR	+	+	+	+	+	+	+
	vasX	-	-	-	+	-	-	-
	vcsC2	+	-	+	-	+	-	-
	vcsN2	+	+	+	-	+	-	-
	vcsV2	+	+	+	-	+	-	-
	vgrG	-	-	-	-	-	-	-
	vspD	+	+	+	-	+	-	-
	zot	-	-	-	-	-	-	-
MGE	***intI1***	-	-	-	+	-	-	-
AMR	***bla-CARB-9***	-	-	+	-	-	-	-
	catB9	-	-	-	-	-	-	-
	drfA1	-	-	-	+	+	-	-
	gyrA	+	+	-	-	-	-	-
	parC	+	+	+	+	+	+	+
	parE	+	+	-	+	+	+	+
	strA	-	-	-	+	-	-	-
	strB	-	-	-	+	-	-	-
	sul2	-	-	-	+	-	-	-
	ICE9_floR	-	-	-	+	-	-	-
Vibrio pathogenicity islands	**VPI-I**	-	-	-	VC0847	-	-	-
	**VPI-II**	VC1758, VC1776	VC1758, VC1776	VC1758, VC1776	VC1790	VC1758, VC1776	VC1758, VC1776, VC1783, VC1790	VC1758, VC1776, VC1783, VC1790
	**VSP-I**	-	-	-	VC0183, VC0185, VC0175, VC0178, VC0180	-	-	-

	**VSP-II**	VC0498	VC0498, VC0490, VC0493	-	-	-	-	-	

Supplementary Table 2: Lineage specific genes identified in the *Vibrio cholerae* genomes

**Row Labels**	**Count of details**
**Core and intermediate**	**1363**
Core: B1+B2 Inter: B3+wave-1+wave-2+wave-3 Rare:	1
Core: B1+B2+B3 Inter: wave-1+wave-2+wave-3 Rare:	324
Core: B1+B2+B3 Inter: wave-2 Rare:	8
Core: B1+B2+B3 Inter: wave-2+wave-3 Rare:	5
Core: B1+B2+B3+wave-1 Inter: wave-2+wave-3 Rare:	15
Core: B1+B2+B3+wave-1+wave-2 Inter: wave-3 Rare:	80
Core: B1+B2+B3+wave-1+wave-3 Inter: wave-2 Rare:	10
Core: B1+B2+B3+wave-2 Inter: wave-1+wave-3 Rare:	346
Core: B1+B2+B3+wave-2+wave-3 Inter: wave-1 Rare:	419
Core: B1+B2+B3+wave-3 Inter: wave-1+wave-2 Rare:	51
Core: B1+B2+B3+wave-3 Inter: wave-2 Rare:	48
Core: B1+B3 Inter: B2+wave-1+wave-2+wave-3 Rare:	1
Core: B1+B3+wave-1+wave-2+wave-3 Inter: B2 Rare:	7
Core: B2+B3 Inter: B1+wave-1+wave-2+wave-3 Rare:	1
Core: B2+B3+wave-1+wave-3 Inter: B1+wave-2 Rare:	6
Core: B3 Inter: B1+B2+wave-1+wave-2+wave-3 Rare:	11
Core: B3+wave-1 Inter: B1+B2+wave-2+wave-3 Rare:	8
Core: B3+wave-1+wave-3 Inter: B1+B2+wave-2 Rare:	2
Core: B3+wave-3 Inter: B1+B2+wave-1+wave-2 Rare:	4
Core: wave-1+wave-2+wave-3 Inter: B1+B2+B3 Rare:	2
Core: wave-2 Inter: B1+B2+B3+wave-1+wave-3 Rare:	2
Core: wave-2+wave-3 Inter: B1+B2+B3+wave-1 Rare:	2
Core: wave-3 Inter: wave-2 Rare:	10
**Core and rare**	**65**
Core: B1+B2+B3 Inter: Rare: wave-2	43
Core: wave-1+wave-2+wave-3 Inter: Rare: B1	21
Core: wave-3 Inter: Rare: wave-2	1
**Core, intermediate and rare**	**33**
Core: B1+B2+B3 Inter: wave-2 Rare: wave-1+wave-3	1
Core: B2 Inter: B1+B3 Rare: wave-3	1
Core: B2+B3 Inter: B1 Rare: wave-3	1
Core: B3+wave-1 Inter: B1+B2+wave-2 Rare: wave-3	2
Core: wave-1 Inter: wave-2+wave-3 Rare: B1	2
Core: wave-1+wave-2 Inter: wave-3 Rare: B1	2

Core: wave-1+wave-3 Inter: wave-2 Rare: B1	2
Core: wave-2 Inter: wave-1+wave-3 Rare: B1	1
Core: wave-2+wave-3 Inter: wave-1 Rare: B1	15
Core: wave-3 Inter: B1+B2+wave-2 Rare: wave-1	3
Core: wave-3 Inter: wave-1+wave-2 Rare: B1	3
**Grand Total**	**1461**

B1: O139 Wave-A, B2: O139 Wave-B, B3: O139 Wave-C, wave -1: O1 wave-1, wave-2: O1 wave-2, wave-3: O1 wave-3.
