# Supplementary material for: Vibrio cholerae O139 genomes provide a clue to why it may have failed to usher in the eighth cholera pandemic
Source: Nat Commun. 2022 Jul 5;13:3864. doi: 10.1038/s41467-022-31391-4 (PMC9256687; doi:10.1038/s41467-022-31391-4)
Supplement: Supplementary file 3 — Description of Additional Supplementary Files [file 41467_2022_31391_MOESM3_ESM.pdf]

Description of Additional Supplementary Files

File Name: Supplementary Data 1
Description: List of Accession IDs of *Vibrio cholerae* genomes used in this study
